# The Public Health and Policy Implications of Epigenetics and Pediatric Health Research

**DOI:** 10.1289/ehp.1205956

**Published:** 2012-10-01

**Authors:** Nsedu Obot Witherspoon, Kristie Trousdale, Cynthia F. Bearer, Rachel L. Miller

**Affiliations:** 1 Executive Director, Children’s Environmental Health Network, Washington, DC, E-mail: nobot@cehn.org; 2 Children’s Environmental Health Network, Washington, DC; 3 University of Maryland School of Medicine, Baltimore, Maryland; 4 Columbia University Medical Center, New York, New York

Despite considerable attention to the field of epigenetic regulation in the scientific literature and its heightened profile at scientific conferences and workshops, the unique connections between epigenetics and pediatric environmental health have not been given specific focus in public meetings. The Children’s Environmental Health Network (CEHN) convened a research conference, The Contribution of Epigenetics in Pediatric Environmental Health, on 30 May–1 June 2012 in San Francisco, California, to address this increasingly important research. CEHN is a national nonprofit organization celebrating 20 years of vital work to protect the developing child from environmental hazards by promoting safer and healthier environments (CEHN 2012).

The conference successfully fostered collaborations across various scientific disciplines to further the understanding of the role of epigenetic regulation in pediatric environmental health, identified research gaps, and explored the relationship of pediatric environmental exposures with epigenetic regulation. The conference also laid the groundwork for translating research findings on epigenetic regulation important to pediatric health outcomes and informing the scientific and public health community about this rapidly growing field.

Key leaders in pediatric and epigenetic research presented their work and actively participated in discussions (see abstracts online; CEHN Research Conference 2012). Much of the research focused on DNA methylation, an important mechanism of environmental influence on gene expression. The scientists addressed important research parameters, including critical windows of exposure, possible differences in epigenetic profiles between boys and girls, and changes in epigenetic markers as children age. Their research covered a range of environmental exposures associated with adverse health effects in children, including alcohol, bisphenol A (BPA), polycyclic aromatic hydrocarbons, arsenic, lead, organochlorines, polybrominated diphenyl ethers, secondhand smoke, and indoor allergens. Findings from a mix of animal and human subject studies generated stimulating discussions of how these exposures affect genes that code for inflammation, asthma, growth, obesity, cognitive development, hormone receptors, and tumor suppression, among others. Several researchers presented results from their work evaluating the role of nutrients such as choline and folate as modifiers of epigenetic changes with regard to specific environmental exposures such as alcohol or arsenic (CEHN Research Conference 2012).

A total of 170 participants—scientists, health care workers, public policy advocates, and educators—participated in the conference. Young investigators were prominent, with 31 student and postdoctoral researchers in attendance. Three Junior Investigator Abstract Awards were presented for outstanding research in the areas of epigenetic effects of prenatal BPA exposure (Marija Kundakovic), DNA methylation and infant growth (Carolyn E. Banister), and epigenetic regulation after environmental intervention (Stephanie Lovinsky-Desir). The international research community was represented by colleagues from Malaysia, Italy, Brazil, India, the Netherlands, Canada, Australia, United Kingdom, South Africa, Austria, France, and China. Federal agencies were represented by participants from the U.S. Environmental Protection Agency, the National Institute of Environmental Health Sciences, and the *Eunice Kennedy Shriver* National Institute of Child Health and Human Development. Media representatives from *Epigenie, New Scientist*, and *Environmental Health Perspectives* were also present.

## Policy Implications

Since its first scientific meeting, sponsored in 1993, CEHN has focused on connecting science with the national pediatric research agenda and with public policy. The 2012 conference was a continuation of that 20-year-long commitment, encouraging the inclusion of the extraordinary field of epigenetics as it relates to pediatric exposures and health.

A unique aspect of this conference was the inclusion of core discussions on the public health and policy implications of epigenetics research in pediatric environmental health. In addition to discussions that followed many of the scientific sessions, a capacity crowd gathered during an early-morning session to discuss policy considerations related to conference topics. The final conference panel also offered a platform for participants to discuss effective ways to take the research presented at the conference as well as the research that continues to emerge and move it into public action. Key questions that participants considered included “How do we move this field into understanding a new pediatric research agenda?” “How do we, collectively, move epigenetic research into policy?” and “How do we communicate with audiences with whom we traditionally do not communicate?”

The robust and passionate conversations during these policy sessions produced key recommendations, as follows.

***Pediatric research agenda recommendations.*** Joseph Weimels, a policy session panelist, provided recommendations for future research to aid policy decisions.

Scientists need to define the normal status of epigenetics in study populations, rather than just observing differences between cases and controls.
Most current research focuses on DNA methylation and genotyping. Proper sample preservation methods need to be used so that researchers can consider other epigenetic markers such as histone modification and miRNA profile changes.
Disease states may develop because our epigenetic state is mismatched to our environment. For instance, our epigenetic state might be well suited to our *in utero* environment but not a match for our adult environment, which could lead to a disease state. Researchers designing a project should both identify the match and mismatch of interest, and consider how and where environmental intervention is possible to reduce the burdens of disease due to the mismatch.

Several researchers suggested that more studies should analyze not only blood and buccal cells, but also tissue cells derived from target organs; that more *in vivo* studies need to assess the stability of epigenetic markers over time; and that behavioral experience, positive or negative, can significantly modify the influence of chemical exposures on neurodevelopment. Research models need to incorporate multiple and combined exposures.

***Policy recommendations.*** Amy Kyle stressed that to effectively influence policy decisions, researchers need to consider which health outcomes our current chemical policies address.

We should utilize a Health in All Policies approach, whose goal is to “help decision-makers understand the links between policies and interventions, health determinants and the resulting health outcomes in a wide range of focus areas” (American Public Health Association 2012), an approach that is understood as a prudent investment for policy makers.
We need to move from the current risk assessment mind-set to the health impact assessment (Centers for Disease Control and Prevention 2012) mind-set wherein cumulative health risks and benefits for all proposed plans, projects, and policies are considered before implementation.
We must design products that are safer, and set the criteria to define and communicate that level of safety.

***Communicating with external audiences.*** Kyle also discussed the challenge of communicating sophisticated epigenetics scientific methods. The older research system was represented by an agent and an exposure, an outcome, and a dose–response curve. The association between the exposure and the health outcome was relatively easy to see. Emerging scientific methods that show small parts of a biological response to gene–environment interactions require skilled translation and outreach efforts.

Scientific advances provide increasing evidence with which to engage public health professionals and policy makers in discussions of pediatric environmental health. From these it is essential to craft one key message that scientists, physicians, and advocates can rally around.
Key scientific information should be more accessible to the public via enhanced open communication across scientific disciplines. Communication with audiences such as health care providers, parents, youth, grandparents, media, policy makers, and public health advocates must clearly identify what is known and what is not known so that they can make informed decisions and speak for their own needs. The many health care practitioners and clinicians in attendance expressed great interest in CEHN’s pediatric training manual (CEHN 1999), which is being updated for publication in 2013 and will continue to serve as a core resource for health care providers and pediatric environmental health curriculum development.
Continued support should be provided for community-based participatory research approaches that bring the advocacy community into basic decision making about what research questions should be posed and answered, and how. Although treatment is important, prevention of disease is our common goal. Kyle emphasized that balanced policy requires balanced, democratic participation, where all stakeholders are engaged.

This conference has led directly to new partnership opportunities for CEHN, specifically providing translation around key findings in pediatric research. We are continuing the conversations with our conference participants, and welcome various ways that our organization can help communicate important peer-reviewed science to the public. We look forward to highlighting the next exciting frontier in children’s environmental health research within the next few years. Richard Jackson, during the final policy session, encapsulated the ultimate goal of environmental and epigenetic research in pediatric health: “Every child has the right to grow up in a safe and healthy environment that is at least as beautiful as the one each of us were given, but hopefully better.”
